# Sleep Duration and Stress Level in the Risk of Gastric Cancer: A Pooled Analysis of Case-Control Studies in the Stomach Cancer Pooling (StoP) Project

**DOI:** 10.3390/cancers15174319

**Published:** 2023-08-29

**Authors:** Giulia Collatuzzo, Claudio Pelucchi, Eva Negri, Manolis Kogevinas, José María Huerta, Jesus Vioque, Manoli García de la Hera, Shoichiro Tsugane, Gerson Shigueaki Hamada, Akihisa Hidaka, Zuo-Feng Zhang, M. Constanza Camargo, Maria Paula Curado, Nuno Lunet, Carlo La Vecchia, Paolo Boffetta

**Affiliations:** 1 Department of Medical and Surgical Sciences, University of Bologna, 40138 Bologna, Italyeva.negri@unibo.it (E.N.); 2 Department of Clinical Sciences and Community Health, Branch of Medical Statistics, Biometry and Epidemiology “G.A. Maccacaro”, University of Milan, 20133 Milan, Italy; claudio.pelucchi@unimi.it (C.P.); carlo.lavecchia@unimi.it (C.L.V.); 3 Consortium for Biomedical Research in Epidemiology and Public Health (CIBERESP), 28029 Madrid, Spain; manolis.kogevinas@isglobal.org (M.K.); vioque@umh.es (J.V.); manoli@umh.es (M.G.d.l.H.); 4 Barcelona Institute for Global Health—ISGlobal, 08036 Barcelona, Spain; 5 IMIM (Hospital del Mar Medical Research Institute), 08003 Barcelona, Spain; 6 Department de Ciències Experimentals i de la Salut, Universitat Pompeu Fabra (UPF), 08003 Barcelona, Spain; 7 Department of Epidemiology, Murcia Regional Health Council, IMIB-Arrixaca, 30120 Murcia, Spain; 8 Instituto de Investigación Sanitaria y Biomédica de Alicante, Universidad Miguel Hernandez (ISABIAL-UMH), 03010 Alicante, Spain; 9 Epidemiology and Prevention Group, Center for Public Health Sciences, National Cancer Center, Tokyo 104-0045, Japan; 10 National Institute of Biomedical Innovation, Health and Nutrition, Tokyo 566-0002, Japan; 11 Nikkei Disease Prevention Center, São Paulo 13010-111, Brazil; 12 Department of Epidemiology, UCLA Fielding School of Public Health and Jonsson Comprehensive Cancer Center, Los Angeles, CA 90095, USA; zfzhang@ucla.edu; 13 Division of Cancer Epidemiology and Genetics, National Cancer Institute, National Institutes of Health, Rockville, MD 20850, USA; 14 Centro Internacional de Pesquisas, A.C.Camargo Cancer Center, São Paulo 01509-010, Brazil; 15 EPIUnit—Instituto de Saúde Pública da Universidade do Porto, 4050-091 Porto, Portugal; nlunet@med.up.pt; 16 Laboratório para a Investigação Integrativa e Translacional em Saúde Populacional (ITR), 4050-600 Porto, Portugal; 17 Departamento de Ciências da Saúde Pública e Forenses e Educação Médica, Faculdade de Medicina da Universidade do Porto, 4200-450 Porto, Portugal; 18 Stony Brook Cancer Center, Stony Brook University, Stony Brook, NY 11794, USA

**Keywords:** gastric cancer, sleep, sleep duration, stress, circadian rhythm, lifestyle, cardia, noncardia

## Abstract

**Simple Summary:**

To our knowledge, this is the first study to address both sleep duration and psychological stress in association with GC risk. Long sleep was associated with gastric cancer, and its subsites and histological types, and stress increased the risk of noncardia cancer in particular. The two exposures exerted an independent effect on GC. These findings contribute to providing evidence for the role of sleep and stress in gastric cancer epidemiology.

**Abstract:**

The association between sleep and stress and cancer is underinvestigated. We evaluated these factors in association with gastric cancer (GC). Five case-control studies from the Stomach Cancer Pooling (StoP) Project were included. We calculated the odds ratios (ORs) and the corresponding 95% confidence intervals (CIs) for sleep duration and stress level in association with GC through multiple logistic regression models adjusted for several lifestyle factors. The analysis included 1293 cases and 4439 controls, 215 cardia and 919 noncardia GC, and 353 diffuse and 619 intestinal types. Sleep duration of ≥9 h was associated with GC (OR =1.57, 95% CI = 1.23–2.00) compared to 8 h. This was confirmed when stratifying by subsite (noncardia OR = 1.59, 95% CI = 1.22–2.08, and cardia OR = 1.63, 95% CI = 0.97–2.72) and histological type (diffuse OR = 1.65, 95% CI = 1.14–2.40 and intestinal OR = 1.24, 95% CI = 0.91–1.67). Stress was associated with GC (OR = 1.33, 95% CI = 1.18–1.50, continuous). This relationship was selectively related to noncardia GC (OR = 1.28, 95% 1.12–1.46, continuous). The risk of diffuse (OR = 1.32, 95% CI = 1.11–1.58) and intestinal type (OR = 1.23, 95% CI = 1.07–1.42) were higher when stress was reported. Results for the association between increasing level of stress and GC were heterogeneous by smoking and socioeconomic status (*p* for heterogeneity = 0.02 and <0.001, respectively). In conclusion, long sleep duration (≥9 h) was associated with GC and its subtype categories. Stress linearly increased the risk of GC and was related to noncardia GC.

## 1. Introduction

Gastric cancer (GC) is one of the neoplasms that has shown large changes in incidence during recent decades [1]. The identification and treatment of *Helicobacter pylori* (*Hp*), the improved level of hygiene and sanitation, and the decrease in tobacco smoking are among the reasons likely explaining this declining trend, together with better preservation and storage of foods and increased intake of fruits and vegetables [2,3]. A high-risk profile can be individuated in males, older than 65, used to tobacco smoking and heavy alcohol drinking (e.g., >47 mg/day), following a diet rich in red meat and salt and poor in fruit and vegetables, infected with *Hp*, and of low socioeconomic status (SES) [4,5,6,7]. GC can be classified by its anatomical location as cardia or noncardia GC, which present slightly different risk profiles and occur with different rates in different geographical areas [8]. Based on its histology, GC can be mainly distinguished into diffuse and intestinal type [9]. The distinct types of GC show different metastatic behavior and different response to therapies, where diffuse and cardia types have worse prognosis and lower response to therapies [9,10,11,12,13]. Therefore, the identification of the differences between these anatomical and histological types of GC has been the objective of many studies [8,14,15,16,17].

Among potential lifestyle risk factors for cancer, sleep duration and level of stress remain underinvestigated. The meta-analysis by Chen et al. found no particular pattern of risk related to sleep duration, except from a positive relationship between short sleep and cancer in Asians and between long sleep and colorectal cancer [18]. Concerning GC, Papantoniou et al. [19] recently described a positive association in individuals reporting ≥9 h of sleep per night and those reporting ≤5 h, compared to the recommended 8 h, describing a U-shaped curve in risk. No difference was found by anatomical subsite and histological type. Sleep duration and cancer development have been studied in the NIH-AARP Health and Diet Study Cohort [20], where a positive risk of about 30% was reported for short sleeping (5–6 h compared to 7–8 h per night). The association between sleep and GC is not fully understood. Disruption of circadian rhythm, poor quality of sleep, and altered sleep duration play a role in several metabolic and chronic diseases, for example, peptic ulcer disease, obesity, and depression [19]. Short sleep duration has been accounted among the effects of chronic stress and unhealthy emotions; these may as well determine long sleeping hours as a symptom of major depression [19]. Long-lasting psychological stress, which can be accompanied by symptoms such as anxiety, irritability, anger, and depression, can be detrimental to health, as reviewed by Zhang and collaborators [21]. Among the mechanisms underlying the role of stress in tumorigenesis, the authors illustrated increased proinflammatory cytokines, change in the immune system toward a tumor-promoting profile, and microbiota dysbiosis [21].

Given that few studies investigated sleep duration and psychological stress in relation to GC risk, and given their physiological role in daily life, we aimed at describing the contribution of each of these two factors in the risk of GC and its subtypes through a pooled-analysis of five studies from the Stomach Cancer Pooling (StoP) Project Consortium.

## 2. Methods

This study is based on the StoP Project Consortium (http://www.stop-project.org/ accessed on 15 May 2023) [22], which includes 34 case-control or nested-within-cohort studies from 15 countries. This consortium of studies aims at examining the role of several determinants in the etiology of GC through pooled analyses of individual-level data, after central collection and validation of the original datasets. The StoP Project received ethical approval from the University of Milan Review Board (reference 19/15 on 1 April 2015). Detailed information on the overall aims and methods was given elsewhere [23].

We excluded 29 studies, mostly due to lack of information on sleep duration and stress, or because >25% of missing values were present on the exposures or main confounders (tobacco smoking, alcohol drinking, socioeconomic status, dietary salt, and fruit and vegetables intake). Subjects with >10% missing data in the variable in the main regression model were excluded. Five individuals with extreme body mass index (BMI) were also excluded (<15 and >50 kg/m^2^).

Data on the two main exposures were collected through multiple-choice questionnaires and referred to at least 1 year before cancer diagnosis/time of interview, in order to reduce the possibility of reverse causation. Details on the type of questions for each exposure are shown in Supplementary Table S2.

Overall, the sleep duration analysis included five studies [14,15,16,17,18,19,20,21,22,23,24,25,26,27], while stress level analysis included four out of five studies, as one [19] lacked information on this exposure. As sleep duration and psychological stress are to some extent interrelated, we also wanted to analyze in the same model both factors to estimate the marginal effect exerted by one factor when adjusting for the other.

Supplementary Table S2 shows the characteristics of each study, including the main results for the single study analyses.

The analysis included histologically confirmed GC cases; matched controls were selected based on hospital or neighborhood. Information on GC anatomical subsite (cardia vs. noncardia, excluding undetermined sites) and histological type (intestinal vs. diffuse type, excluding undefined histology) were available for all studies.

Self-reported sleep duration was assessed based on the number of hours of night sleep—excluding daily naps. We consequently categorized sleep hours into 4 groups: ≤6, 7, 8, and ≥9 h. We used 8 h of sleep as reference based on the US National Sleep Foundation recommendation [28]. Psychological stress was also derived from the questionnaires, where the participants answered questions such as “how often do you feel under stress which makes you tense or worried, or cause physical problems such as stomach or back trouble or headache?” or “to which level of stress are you exposed in your daily life, including work and family life?”. Then, frequency and intensity were combined to categorize psychological stress as low, moderate, or high level. Data were harmonized according to a prespecified format, and completeness and consistency between variables were carefully checked.

The final regression models included terms for study, sex, age (≤54, 55–59, 60–64, 65–69, ≥70 years), smoking status (never smoker, former smoker, current smoker), socioeconomic status (study-specific low, intermediate, high as defined in each original study based on education, income, and/or occupation), alcohol drinking (overall consumption: never, low—≤12 gr/day, intermediate 13–47 gr/day, high—>47 gr/day), salt consumption (low, intermediate and high, based on study-specific categories), and vegetable and fruit intake (low, intermediate, and high, based on study-specific tertiles). *Hp* status was not included in the analysis given the high number of missing values. For stress level, we estimated the odds ratios (OR) of GC for increasing psychological stress; concerning sleep duration, it could not be treated as a continuous variable because of the lack of linearity in the dose–response relationship (the reference was 8 h of sleep per night).

We conducted stratified analyses by sex, smoking status, SES, anatomical subsite (cardia vs. noncardia), and histological type (intestinal vs. diffuse). We also considered a potential interaction between sleep and stress.

The effect of race/ethnicity (Caucasian/Black/Asian/Hispanic or Latin/others) and family history of GC in first-grade families (yes vs. no) were also explored, but they did not affect the results and were not included in the final models. Similarly, the main model was additionally adjusted for BMI to explore its potential confounding role.

To assess the contribution of individual studies to the overall results, we repeated the pooled analysis within each single study, and excluding one study at the time.

All the statistical analyses were performed on STATA, version 16.1 (Stata Corp., College Station, TX, USA) [29]. A two-tailed *p*-value lower than 0.05 was considered significant.

## 3. Results

### 3.1. Descriptive Analyses

The analysis included 5732 individuals, comprising 1293 GC cases and 4439 controls. The case group included 215 cardia and 919 noncardia GC, along with 353 diffuse and 619 intestinal types. Table 1 shows their distribution by study, sex, age, and major covariables. Cases were more frequently of low socioeconomic status (67.1%) than controls (53.6%) and were more frequently smokers (25.1% vs. 19.6%). Cases reported more often 8 (36.8% vs. 30% of the controls) and ≥9 h of sleep (14.9% vs. 9.1% of the controls), as shown in Table 2. The prevalence of high psychological stress was higher among cases than controls (38.6% vs. 30.5%), while there was no difference for intermediate level (both around 28%); consequently, cases were less likely to declare low stress levels than controls (32.5% vs. 41.0%). Higher stress levels were also reported more frequently by women (42.9%) than men (29.7%), while the proportion was inverted for low stress levels (28.0% vs. 41.9% respectively). Further details are shown in Table 2.

There was no correlation between stress level and short sleep duration (*p* = 0.16 among controls), while a negative correlation was found with long sleep duration (*p* = 0.01).

### 3.2. Analyses of the Association between Sleep Duration and Gastric Cancer

The results of the multivariable analysis on the duration of sleep are shown in Table 3. After multiple adjustment, sleep duration of ≥9 h was associated with GC (OR = 1.57, 95% CI = 1.23–2.00) compared to 8 h. Neither race/ethnicity nor family history of GC affected the results.

Results stratified by anatomical subsite within the stomach and histological type are reported in Table 4. The relationship between sleep duration and GC risk was confirmed when stratifying by anatomical subsite, being the OR of noncardia GC for ≥9 h of sleep equal to 1.56 (95% CI = 0.97–2.77) and that of cardia GC equal to 1.59 (95% CI = 1.22–2.08). The risk by histological types was also similar, with ≥9 h being significantly associated with diffuse (OR = 1.65, 95% CI = 1.14–2.40) and a positive but not significant association with intestinal type (OR = 1.24, 95% CI = 0.91–1.67). Very mild difference was found by sex, with men having an OR of 1.51 (95% CI = 1.11–2.03) and women of 1.74 (95% CI = 1.15–2.64) when reporting ≥9 h of sleep (Table 5). Long sleeping hours increased the risk of GC among individuals aged 65 or more years of age (OR = 1.70, 95% CI = 1.25–2.34), but not the younger ones (OR = 1.37, 95% CI = 0.91–2.07), despite this difference was not statistically significant (*p* for heterogeneity = 0.410). No difference was found by smoking status (*p* for heterogeneity = 0.622). Last, no difference was observed by SES (*p* for heterogeneity = 0.794), despite we observed only a significant association between ≥9 h of sleep and GC in individuals from low level (OR = 1.58, 95% CI = 1.18–2.11).

### 3.3. Analyses of the Association between Stress and Gastric Cancer

When considering stress levels, we included 843 GC cases and 976 controls.

Results of the multivariable analysis are reported in Table 3. Overall, psychological stress was linearly associated with GC, where high vs. low stress level corresponded to OR = 1.77 (95% CI = 1.39–2.26), with 34% of excess risk for 1 level of increase in stress (95% CI = 1.18–1.50). In the analyses stratified by anatomical subsite and histologic type (Table 4), the relationship between stress level and GC risk was found to be present for noncardia GC, for which the OR was 1.28 for one category of increase (95% CI = 1.12–1.46), while no significant association was observed for cardia GC. These figures resulted to be heterogeneous (*p* = 0.01). The risk of diffuse GC increased in individuals reporting high levels of stress compared to low levels (OR = 1.77, 95% CI = 1.24–2.53), with an OR for the continuous analysis equal to 1.32 (95% CI = 1.11–1.58); similar results were found for intestinal type (OR = 1.52, 95% CI = 1.15–2.00) in subjects with high level of stress; OR for the continuous analysis equal to 1.23 (95% CI = 1.07–1.42; *p* for heterogeneity = 0.540).

Despite the different distribution of stress levels in men and women, the stratified analysis did not reveal an effect modification by sex (details are shown in Table 5; *p* for heterogeneity = 0.651). No difference was found by age groups (*p* for heterogeneity = 0.684), while stress effect resulted to be slightly more pronounced among current smokers (OR = 1.82, 95% CI = 1.41–2.36 for current vs. 1.18, 95% CI = 0.99–1.40 for never smokers; *p* for heterogeneity = 0.02). Also, a significant heterogeneity was revealed for stress and GC by level of socioeconomic status (OR = 1.53, 95% CI = 1.30–1.79 in low vs. OR = 0.65, 95% CI = 0.44–0.97 in high SES, *p* for heterogeneity < 0.001).

### 3.4. Sensitivity Analyses

As shown in Supplementary Table S1, sensitivity analyses considering each study singularly indicated that the results are heterogeneous. The most noticeable is the opposite direction of the association between stress level and GC in the US study. Additionally, sensitivity analyses excluding one study at a time (Supplementary Table S3) revealed again some heterogeneity between the studies, with the US study being a clear outlier when compared to the others [24]. Indeed, this study did not reveal any association between sleep and GC, while stress resulted to be inversely related, thus showing an opposite trend with respect to our overall results.

## 4. Discussion

In this pooled analysis, we found a positive association between ≥9 h of sleep duration and GC. High stress level was also associated with GC, with a significant dose–response relationship. We observed the highest risk of GC in the category of long-sleepers (≥9 h), suggesting that the normal/short-sleep pattern is overall more favourable.

These results were confirmed for both major histological GC types, being slightly stronger for the diffuse type. In terms of anatomical subsite, as compared to cardia, noncardia GC showed a stronger association with both sleep duration and stress level.

Stratification analyses showed a stronger effect of sleep duration in women and individuals aged 65 or older; concerning sex and age, no difference was found in the effect exerted by stress, while we observed a particularly increased GC risk in current smokers. Additionally, the effect of stress (but not that of sleep duration) on GC risk in low-socioeconomic-status vs. high-socioeconomic-status individuals showed opposite directions. Subjects from less affluent groups of the population may experience different economic and psychosocial problems than subjects from high social class. While this may explain at least in part the mechanism of reverse causality under the association between stress and GC which we found, this difference was not observed for sleep duration. Regardless, we suggest using this information with caution rather than considering it as definite findings, for which further studies specifically developed to investigate these exposures are needed.

The interaction between sleep duration and stress level were consistent with a multiplicative model, suggesting independent effects on GC risk.

Sleep is fundamental in the biochemical and hormonal homeostasis in humans [30,31]. Quality of sleep is linked to its duration [30,31]. Commonly, sleep duration ranges between 6 and 9 h, despite being determined by daily life events and environmental clues [32]. Indeed, shorter hours of sleep are reported to be associated with several health conditions, from cardiovascular disease to diabetes and cancer [33]. Short sleep duration can indeed negatively influence several homeostatic systems, possibly resulting in procarcinogen effects on stem cells [34,35]. Moreover, the bidirectional relationship between psychological disorders and sleep is well known. For example, subjects with short sleeping patterns (defined as <7 h in general) tend commonly to suffer from anxiety disorder, causing difficulty in maintaining the rest and possibly experiencing lack of sleep, or insomnia [32,36,37]. Short sleep can cause, in turn, different symptoms, including gastrointestinal (GI) tract symptoms [38,39,40], and also increases stress levels. Nevertheless, the temporality of this association is difficult to explain without a longitudinal follow-up. On the other hand, long sleep duration (commonly referred as >8–9 h of sleep) also seems to act negatively on health, and could be a symptom of major depression. Once sleep exceeds 8/9 h, metabolism takes a direction that results in an increased level of carcinogenic factors [35,41]. The association between long sleep duration and GI cancers has been explained as potentially confounded by low SES, low physical activity, and additional comorbidities. While we adjusted for SES level, the other potential confounders were not included in this analysis. Despite this, when adjusting for BMI—the effect of which, according to Chen et al. [42], may be mediated by long sleep hours through the change in hormones involved in appetite regulation—the association between long sleep duration and GC was still confirmed in our study population (OR = 1.73, *p* < 0.001).

A nationwide case-control study conducted in Taiwan on data from 2001–2011 identified a significantly higher cancer risk in individuals with sleep disorders, and specifically breast cancer being linked to insomnia, parasomnia, and obstructive sleep apnea, this latter also causing nasal and prostate cancer in a range of risk between 1.70 and 6.00 [43].

Few studies are available to compare our results on GC. To our knowledge, our findings are consistent with current literature on the topic, despite the relationship between sleep duration and GC is elsewhere described through a U-shaped curve that we do not observe [44]. U-shaped results have been also described for non-neoplastic conditions such as metabolic syndrome [45] and amyloid-beta burden [46]. Sleep duration and its annual changes were examined in a large Chinese cohort in relation to GI-cancers by Chen et al., finding no association with GC [42].

Sleep is a complex vital function that can take different shapes in an individual’s course of life, primarily based on age [46]. Circadian rhythm and sleep patterns can vary based on life circumstances [32], such as employment status and type of job, as well as marital and parental status [47], and can also depend on daily energy consumption [48,49]. Disease itself can change circadian rhythm [50]. Also, there is a large interindividual variability in the duration of sleep. Even if the range of physiological sleeping time is quite large (i.e., 7–9 h) [28], humans suffer from detrimental effects when the duration of sleep goes beyond the limits. Altered sleep duration in either direction appears to determine the same shape of detrimental health effects, including an enhanced risk of cancer. A recent meta-analysis on six population-based cohort studies from Japan described an increased risk of cancer mortality for ≥10 h of sleep, supporting this association and arguing against reverse causality [51]. The available evidence, however, is not fully consistent and sleep dose–response curves should be further studied in relation to health outcomes, including cancer [52]. The direction of the association between sleep disruption and cancer was recently reviewed by Berisha and coauthors, illustrating several potential mechanisms under sleep disruption-induced inflammation and alterations of antitumor immunity [53]. Moreover, the authors address the association of psychological stress and sleep problems: stress promotes the prolonged activation of the hypothalamic–pituitary–adrenocortical axis and the sympathetic–adrenal–medullary system, which becomes a change in the circadian rhythm; sleep disruption can trigger stress response; as a consequence, sleep and stress reciprocally influence one another and often coexist [53]. As mentioned in a review [54], Titova and coauthors reported suggestive findings around the association between genetic variants linked to short sleep duration and certain types of cancer in a Mendelian randomization analysis aimed at investigating the causal nature of the relationship between these two factors [53]. Specifically, a significant higher risk of GC was described in carriers of short-sleep genes. Nonetheless, this analysis was based on multiple comparison and its results require cautious interpretation [34].

In our analysis, high stress level was almost three times more likely to be observed in cases than in controls, with a significant trend in risk. This relationship appeared to be independent from that with sleep duration and GC.

Stress response is accompanied by emotional and physical signs and symptoms, also affecting many GI functions by stimulating secretion and colonic transit, delaying gastric emptying time, increasing intestinal permeability and visceral sensitivity, and modifying intestinal microbiota [55].

Stress response is physiological in human life and occurs every time a person faces an event that disrupts his or her balance, and constitutes a menace [56]. Stress is not always a negative factor, as it can drive positive behavior that in turn is translated in the organism into healthy biochemical signals [57]. On the contrary, chronic stress realizes a condition that facilitates the occurrence of diseases, making the organism more vulnerable and also creating a proinflammatory microenvironment that can contribute to tumorigenesis and cancer progression [58]. Cancer is a stressful life event that strongly impacts sleep quality, making difficult the distinction of a causal role in retrospective studies [54]. Regardless, evidence for a stressful life event preceding the occurrence of certain types of cancer has been provided [54].

The exposure investigated in the present analysis addresses psychological stress, combining both intensity and frequency; the resulting variable was operationalized in three categories of increasing stress, ranging from low level (corresponding to the lowest frequency, lowest intensity declared) to high level (most frequently reported and with highest intensity).

To our best knowledge, this is the first study that simultaneously analyzed the risk of GC in association with both sleep and stress. We performed several sensitivity analyses that confirmed the main results, consistently showing an increased risk of GC in individuals with long sleep duration and high level of stress. These effects appeared not to depend on reciprocal confounding or effect modification. Based on our knowledge, this analysis is the first that tried to unravel the effects of sleep from that of psychological stress on GC.

The disease can have partially influenced the two exposures. That is, GC cases may have more often experienced altered sleep duration and high level of stress compared with controls because of potential symptoms. Indeed, GC patients are often burdened by sleep disorders, poor quality of sleep, and high levels of anxiety. This finds confirmation in a recent case-control study, where sleep disorder and risk of GC were significantly associated only within 1 year prior to cancer diagnosis and not when sleep disorders were documented earlier. In our pooled analysis, the information collected with questionnaires referred to the year before cancer diagnosis/interview, i.e., to a period when GC patients were likely to be asymptomatic.

This analysis is impaired by some limitations. The main problem concerns the possibility of reverse causation when examining the two exposures we investigated. Indeed, the case-control design of the included studies could not eliminate the possibility of selection bias and recall bias, and that of reverse causation, despite the cases were asked about their previous (at least 1 year before diagnosis) sleep behaviors. The data reported may be affected by recall bias, given the time lag between the requested exposure and the questionnaire administration. Moreover, we cannot exclude that altered sleep or psychological stress presented as early symptoms of GC in this timeframe. As pointed out in previous studies [18], exposure assessment should be performed by using reliable instruments, such as wearable devices able to measure sleep quality and duration. Regardless, this would not be possible for stress levels, which remain a subjectively reported factor.

The included studies were not designed to investigate these exposures. Also, *Hp* status was not considered in this analysis because of missing values in the participating studies. This is a potential limitation when focusing on stress levels, as far as *Hp* can cause gastric and extra-gastric symptoms which may have a psychological burden. Thus, stress can mediate part of the *Hp* effect. Four out of five of the studies included in this analysis were hospital-based, impairing the generalizability of the data and implying the collected information on sleep and stress, which are largely dependent on condition of the subject, may have been affected by selection bias and recall bias. Despite this, the questions referred to usual habits, thus outside the hospitalization period.

The large sample size of our analysis, the detailed sociodemographic and lifestyle information available for study subjects, and the pathologic confirmation of case diagnoses are strengths of our study.

## 5. Conclusions

Our study provides evidence for a positive association of GC with both long hours of sleep and high stress levels. The two exposures resulted to be independently related to GC. These associations were confirmed for both intestinal and diffuse types, and cardia and noncardia subsites of GC. Neuropsychological factors such as sleep characteristics and stress level would need more investigation in relation to neoplastic disease and specifically GC. Prospective studies designed to investigate these exposures would be warranted to clarify their role in cancer epidemiology and specifically GC epidemiology.

## Figures and Tables

**Table 1:** Distribution of cases of GC and controls according to study center, sex, age, and selected characteristics *. The StoP consortium.

Characteristics	Cases	Controls
	N (%)	N (%)
Total	1293 (100.0)	4439 (100.0)
Sex		
Male	871 (25.7)	2518 (74.3)
Female	422 (18.0)	1921 (82.0)
Age (years)		
<55	286 (22.2)	1000 (77.8)
55–59	160 (25.0)	480 (75.0)
60–64	163 (19.6)	667 (80.4)
65–69	237 (23.7)	809 (77.3)
≥70	447 (23.2)	1483 (76.8)
Sleep duration (hours)		
<6 h	356 (26.3)	1354 (73.7)
7 h	250 (20.8)	1131 (79.2)
8 h	476 (18.1)	1332 (81.9)
≥9 h	192 (32.3)	402 (67.7)
Stress level		
Low	274 (59.4)	400 (40.7)
Intermediate	244 (53.3)	278 (46.7)
High	325 (47.8)	298 (52.2)
Cigarette smoking		
Never	549 (21.4)	2018 (78.6)
Former	409 (21.5)	1491 (78.5)
Current	324 (27.1)	871 (72.3)
Alcohol drinking †		
Never	476 (30.7)	1074 (69.3)
Low	312 (16.8)	1548 (83.2)
Intermediate	247 (21.4)	905 (78.6)
High	143 (37.2)	241 (62.8)
Socioeconomic status		
Low	867 (26.7)	2381 (73.3)
Intermediate	293 (19.2)	1235 (80.8)
High	131 (13.8)	818 (86.2)
Salt intake		
Low	599 (26.5)	1664 (73.5)
Intermediate	216 (15.9)	1145 (84.1)
High	363 (24.5)	1116 (75.5)
Vegetables and fruit intake		
Low	310 (22.0)	1102 (78.0)
Intermediate	320 (21.8)	1147 (78.2)
High	548 (26.5)	1524 (73.6)
Study population	NA	
Hospitalized		891 (21.1%)
Nonhospitalized		13,328 (79.9%)
Anatomical site of GC		NA
Cardia	215	
Noncardia	919	
Histological type of GC		NA
Intestinal	619	
Diffuse	353	

* Numbers may not add to the total because of missing values. † Categorized based on alcohol consumption as follows: never; low, ≤12 gr/day; intermediate, 13–47 gr/day; high, >47 gr/day. GC, gastric cancer; NA, not applicable.

**Table 2:** Characteristics of the study population by sleep duration and stress level. The StoP consortium.

	Sleep Duration				Stress Level		
	≤6 h	7 h	8 h	≥9 h	Low	Intermediate	High
Sex							
Male	56.7%	59.4%	60.0%	62.5%	73.9%	64.9%	55.7%
Female	43.3%	40.6%	40.0%	37.5%	26.1%	35.1%	43.3%
Age							
<55	21.6%	23.5%	28.3%	11.6%	18.3%	28.7%	32.4%
55–59	11.1%	11.9%	12.7%	6.73%	9.94%	16.1%	14.6%
60–64	14.4%	14.4%	16.2%	11.6%	14.8%	16.1%	16.0%
65–69	19.5%	18.4%	16.4%	19.0%	20.9%	19.4%	18.0%
≥70	33.4%	31.9%	26.4%	51.0%	35.1%	19.7%	19.0%
Cigarette smoking							
Never	44.9%	42.1%	47.0%	50.8%	43.1%	50.7%	48.8%
Former	34.7%	35.7%	30.2%	32.6%	33.1%	28.0%	25.8%
Current	20.4%	22.2%	22.8%	16.6%	23.8%	21.3%	25.5%
Alcohol drinking							
Never	33.3%	23.4%	33.8%	35.9%	50.5%	60.4%	48.7%
Low	39.8%	44.3%	34.0%	27.7%	20.6%	16.2%	24.2%
Intermediate	21.0%	24.5%	23.5%	26.6%	18.3%	14.8%	17.5%
High	5.90%	7.80%	8.70%	9.87%	10.6%	8.65%	9.60%
Socioeconomic status							
Low	55.7%	46.3%	58.0%	73.4%	61.4%	62.8%	62.8%
Intermediate	27.7%	29.9%	26.1%	20.2%	26.4%	27.6%	24.2%
High	16.6%	23.8%	15.8%	6.40%	12.2%	9.6%	13.0%
Vegetables and fruit intake							
Low	27.8%	31.2%	27.6%	26.1%	19.7%	18.8%	24.8%
Intermediate	28.2%	32.0%	29.6%	28.8%	20.6%	20.0%	26.4%
High	44.0%	36.8%	42.8%	45.1%	59.6%	61.2%	48.8%
Anatomical site of GC							
Cardia	19.8%	19.7%	17.9%	19.0%	21.6%	12.0%	16.2%
Noncardia	80.2%	80.3%	82.1%	81.0%	78.4%	88.0%	87.8%
Histological type of GC							
Intestinal	61.8%	60.8%	66.0%	64.4%	70.1%	60.0%	62.5%
Diffuse	38.2%	39.2%	34.0%	35.6%	29.9%	40.0%	37.5%

GC, gastric cancer.

**Table 3:** Odds ratio of gastric cancer for sleep duration and stress level. The StoP consortium.

	OR1 (95% CI)	OR2 (95% CI)
Sleep duration (hours)		
≤6 h	0.97 (0.80–1.18)	0.99 (0.77–1.28)
7 h	0.86 (0.70–1.05)	1.03 (0.79–1.35)
8 h	Ref	Ref
≥9 h	1.57 (1.23–2.00)	1.60 (1.14–2.23)
Stress level		
Low	Ref	Ref
Intermediate	1.40 (1.10–1.81)	1.44 (1.12–1.85)
High	1.77 (1.39–2.26)	1.82 (1.42–2.32)
Ordinal *	1.33 (1.18–1.50)	1.35 (1.19–1.52)

OR1, odds ratio adjusted for study, sex, age, smoking status, alcohol drinking, socioeconomic status, salt intake, vegetable and fruit intake. OR2, odds ratio, additionally adjusted for stress level (analysis of sleep duration) and sleep duration (analysis of stress level). CI, confidence interval; Ref, reference category. * Odds ratio for increase in one level of stress.

**Table 4:** Odds ratio of gastric cancer for sleep duration and stress level by anatomical site and histological type. The StoP consortium.

	Anatomical Subsite		Histological Type	
	Cardia	Noncardia	Diffuse	Intestinal
	OR (95% CI)	OR (95% CI)	OR (95% CI)	OR (95% CI)
Sleep duration (hours)				
≤6 h	0.93 (0.61–1.41) N = 63	0.96 (0.77–1.19) N = 256	0.99 (0.73–1.35) N = 99	1.00 (0.78–1.28) N = 160
7 h	0.80 (0.51–1.24) N = 43	0.86 (0.68–1.09) N = 175	0.95 (0.68–1.33) N = 71	0.84 (0.64–1.10) N = 110
8 h	Ref N = 72	Ref N = 331	Ref N = 126	Ref N = 245
≥9 h	1.63 (0.97–2.72) N = 34	1.59 (1.22–2.08) N = 145	1.65 (1.14–2.40) N = 52	1.24 (0.91–1.67) N = 94
Stress level				
Low	Ref N = 53	Ref N = 192	Ref N = 66	Ref N = 155
Intermediate	0.92 (0.52–1.62) N = 26	1.45 (1.10–1.90) N = 190	1.49(1.03–2.15) N = 82	1.22 (0.91–1.63) N = 123
High	0.79 (0.46–1.36) N = 39	1.62 (1.24–2.13) N = 201	1.77 (1.24–2.53) N = 100	1.52 (1.15–2.00) N = 167
Ordinal *	0.89 (0.68–1.17)	1.28 (1.12–1.46)	1.32 (1.11–1.58)	1.23 (1.07–1.42)

OR, odds ratio, CI, confidence interval; Ref, reference category. N = number of cases. Adjusted for study, sex, age, smoking status, alcohol drinking, socioeconomic status, salt intake, vegetable and fruit intake. * Odds ratio for increase in one level of stress. *p* heterogeneity for sleep ≥ 9, cardia vs. noncardia: 0.933, diffuse vs. intestinal: 0.244, *p* heterogeneity for stress (continuous), cardia vs. noncardia: 0.01, diffuse vs. intestinal: 0.540.

**Table 5:** Odds ratios of gastric cancer for sleep duration and stress level, by sex, age, and smoking status. The StoP consortium.

	Sex		Age		Cigarette Smoking			Socioeconomic Status	
	OR (95% CI)		OR (95% CI)		OR (95% CI)			OR (95% CI)	
	*Male*	*Female*	*<65 years old*	*≥65 years old*	*Never*	*Former*	*Current*	*Low*	*High*
Sleep duration (hours)									
≤6 h	1.00 (0.80–1.27) N = 319	0.88 (0.62–1.23) N = 157	0.87 (0.66–1.15) N = 234	1.06 (0.81–1.40) N = 242	0.97 (0.73–1.30) N = 210	0.74 (0.52–1.05) N = 140	1.26 (0.83–1.93) N = 125	1.11 (0.86–1.43) N = 233	0.59 (0.32–1.08) N = 35
7 h	0.84 (0.66–1.08) N = 237	0.84 (0.58–1.22) N = 119	0.75 (0.56–1.01) N = 181	1.00 (0.75–1.35) N = 175	0.82 (0.59–1.13) N = 149	0.66 (0.46–0.96) N = 118	1.18 (0.78–1.79) N = 82	0.87 (0.67–1.15) N = 151	0.73 (0.41–1.30) N = 41
8 h	Ref N = 180	Ref N = 70	Ref N = 126	Ref N = 124	Ref N = 88	Ref N = 87	Ref N = 74	Ref N = 323	Ref N = 44
≥9 h	1.51 (1.11–2.03) N = 124	1.74 (1.15–2.64) N = 68	1.37 (0.91–2.07) N = 62	1.70 (1.25–2.32) N = 130	1.46 (1.02–2.07) N = 92	1.34 (0.86–2.08) N = 59	1.89 (1.09–3.28) N = 39	1.58 (1.18–2.11) N = 145	1.37 (0.49–3.84) N = 10
Stress level									
Low	Ref N = 209	Ref N = 65	Ref N = 108	Ref N = 166	Ref N = 113	Ref N = 92	Ref N = 67	Ref	Ref
Intermediate	1.75 (1.28–2.37) N = 175	0.97 (0.61–1.53) N = 69	1.41 (1.00–1.99) N = 142	1.39 (0.96–2.03) N = 102	1.13 (0.79–1.63) N = 107	1.69 (1.05–2.72) N = 77	1.84 (1.06–3.18) N = 57	1.70 (1.23–2.35) N = 169	0.54 (0.23–1.27) N = 39
High	1.86 (1.37–2.53) N = 193	1.62 (1.07–2.46) N = 132	1.75 (1.26–2.44) N = 193	1.94 (1.34–2.82) N = 103	1.38 (0.97–1.97) N = 140	1.75 (1.10–2.80) N = 83	3.32 (1.98–5.56) N = 100	2.32 (1.70–3.18) N = 268	0.43 (0.20–0.96) N = 17
Ordinal *	1.38 (1.19–1.61)	1.30 (1.05–1.60)	1.32 (1.12–1.56)	1.39 (1.16–1.68)	1.18 (0.99–1.40)	1.34 (1.06–1.69)	1.82 (1.41–2.36)	1.53 (1.30–1.79) N = 220	0.65 (0.44–0.97) N = 28

OR, odds ratio; CI, confidence interval; Ref, reference category. N = number of cases. Adjusted for study, sex, age, smoking status, alcohol drinking, socioeconomic status, salt intake, vegetable and fruit intake. * Odds ratio for increase in one level of stress. *p* heterogeneity for sleep ≥ 9, men vs. women: 0.588; <65 years old vs. ≥65 years old: 0.410; never vs. former vs. current smoker: 0.622; low vs. high socioeconomic status: 0.794; *p* heterogeneity for stress (continuous), men vs. women: 0.651; <65 years old vs. ≥65 years old: 0.684; never vs. former vs. current smoker: 0.02; low vs. high SES: <0.001.

## Data Availability

Data can be obtained from the StoP Project according to the provisions established by the Consortium (stop-project.org). Further information is available from the corresponding author upon request.

## Supplementary Materials

The following supporting information can be downloaded at: https://www.mdpi.com/article/10.3390/cancers15174319/s1, Table S1. Selected characteristics of the studies included in the pooled analysis, and study-specific odds ratio of gastric cancer for sleep duration and stress level; Table S2. Available information on the data collection on sleep and stress; Table S3. Adjusted Odds ratio of gastric cancer for sleep duration and stress level—Results of pooled analysis excluding one study at a time.
