# Supplementary material for: Sleep Duration and Stress Level in the Risk of Gastric Cancer: A Pooled Analysis of Case-Control Studies in the Stomach Cancer Pooling (StoP) Project
Source: Cancers (Basel). 2023 Aug 29;15(17):4319. doi: 10.3390/cancers15174319 (PMC10486543; doi:10.3390/cancers15174319)
Supplement: Supplementary file 1 [file cancers-15-04319-s001.zip › cancers-2482754-supplementary.pdf]

Supplementary Table S1. Selected characteristics of the studies included in the pooled analysis, and study-specific odds ratio of gastric cancer for sleep duration and stress level.

Reference	Country	Study period	Cases (n)	Controls (n)	Study design	Exposures investigated	OR, 95% CI Sleep duration*	OR, 95% CI Stress level†
Zhang et al, 1999 [24]	New York, USA	1992-1994	132	132	Hospital-based	Sleep, stress	0.82, 0.40-1.66<br>1.08, 0.50-2.34<br>1.05, 0.28-3.89	0.22, 0.09-0.58<br>0.34, 0.18-0.66
Papantonious et al., 2021 [19]	10 provinces, Spain	2008-2012	441	3440	Population-based	Sleep	0.82, 0.61-1.11<br>0.59, 0.41-0.84<br>1.36, 0.95-1.97	NA
Santibanez et al., 2012 [25]	Valencia, Spain	1995-1999	401	455	Hospital-based	Sleep, stress	1.61, 1.01-2.57<br>1.16, 0.80-1.69<br>2.27, 1.43-3.59	1.62, 1.10-2.37<br>3.30, 2.31-4.73
Nashimoto et al.,2002 [26]	Sao Paulo, Brazil (A)	1991-1994	226	226	Hospital-based (Brazilian residents)	Sleep, stress	0.80, 0.50-1.28<br>0.88, 0.47-1.62<br>0.69, 0.35-1.38	2.03, 1.22-3.37<br>1.72, 1.01-2.93
Hamada et al., 2002 [27]	Sao Paulo, Brazil (B)	1991-1994	93	186	Hospital-based (Japanese residents)	Sleep, stress	0.67, 0.35-1.27<br>0.67, 0.29-1.55<br>1.49, 0.62-3.61	1.14, 0.59-2.21<br>1.75, 0.82-3.76

OR, odds ratio, adjusted for sex, age, smoking status, alcohol drinking, socioeconomic status, salt intake, vegetable and fruit intake
CI, confidence interval; NA, not available
* OR for ≤6, 7, and ≥9 (Reference category: 8 hours)
† OR for intermediate and high level (Reference category: low level)

Supplementary Table S2. Available information on the data collection on sleep and stress.

Study reference	Sleep assessment	Stress assessment	Selection of cases and controls	When do the questions refer to	Validation
Hamada et al., 2002 [27]	Questionnaire	Questionnaire	Cases were pure Japanese ancestry newly diagnosed with gastric cancer in one of 13 collaborating hospitals for treatment in the city of São Paulo. Controls were identified from among the inpatients of the same hospitals. In only one hospital, a specialized cancer institution, the controls were selected from neighboring public hospitals. However, it was not possible to achieve the desired matching within the hospital, and 80 of 192 control patients were recruited voluntarily from the Japanese community in São Paulo.	1991-1994	No
Nishimoto et al., 2002 [26]	Questionnaire	Questionnaire	Cases were 250 Brazilian patients from 13 collaborating hospitals, of non-Japanese origin. Controls were identified from among the inpatients of the same hospitals. In only one hospital, a specialized cancer institution, the controls were selected from neighboring public hospitals.	1991-1994	No
Santibanez et al., 2012 [25]	Questionnaire administered the year before Dx.<br>Self-reported usual # of hours	Questionnaire administered the year before Dx.<br>Self-reported usual level of stress: How often do you feel under stress	Hospital-based. Identification of incident cases (New Dx of Gastric cancer). Controls were frequency matched to the expected distribution<br>of case subjects in the overall PANESOES study by age group	Questions referred to 1-5 years before the diagnosis for cases (or interview date for controls)	No. Questions were chosen from other questionnaires (SCIRO study)

	of sleep per day:<br>1 <=6; 2, 7 h; 3, 8h/d; 4 9+ h/d	1. daily<br>2. several x week<br>3. Several x month<br>4. several x year<br>5. never	(,60, 60–70 and .70 years), sex and province (Alicante and<br>Valencia). A wide inclusion criterion was used to select controls<br>with diseases not related a priori to the main exposures of<br>interest (tobacco, alcohol and diet).		
Papantoniou et al., 2021 [19]	Evaluated at recruitment. Sleep duration: average number of hours/night; frequency (days/week) of daily naps ("siestas"), and their average duration (minutes/nap).	-	Population-based multicase-control study (MCC-Spain). Cases and controls from 12 Spanish provinces (Asturias, Barcelona, Cantabria, Girona, Granada, Gipuzkoa, Huelva, León, Madrid, Murcia, Navarra and Valencia). Recruitment was simultaneous. All incident cancer cases (histologically confirmed, with no prior history of cancer) from 23 participating hospitals were invited to join; controls were randomly selected from administrative records of primary health centers within the hospital's catchment areas and frequently matched to the pool of cases, by age, sex, and region.	Questions on sleep habits referred to the last 10 years. Sleep problems were considered for periods >=1 year throughout lifetime.	No. Literature review and ad hoc questions.

Supplementary Table S3. Adjusted Odds ratio of gastric cancer for sleep duration and stress level – Results of pooled analysis excluding one study at a time.

Excluded study	OR, 95% CI Sleep duration*	OR, 95% CI Stress level†
Zhang et al, 1999 [24]	0.99, 0.81-1.21	1.73, 1.32-2.26
	0.84, 0.67-1.04	2.45, 1.87-3.20
	1.60, 1.25-2.04	
Castano-Vinyalis et al., 2015 [10]	1.05, 0.82-1.36	NA
	1.07, 0.82-1.40	
	1.54, 1.11-2.14	
Santibanez et al., 2012 [25]	0.84, 0.67-1.04	1.16, 0.83-1.64
	0.73, 0.56-0.93	1.01, 0.72-1.42
	1.25, 0.93-1.68	
Nashimoto et al.,2002 [26]	0.98, 0.79-1.22	1.20, 0.89-1.62
	0.84, 0.67-1.05	1.83, 1.39-2.41
	1.74, 1.34-2.25	
Hamada et al., 2002 [27]	1.00, 0.81-1.22	1.43, 1.09-1.88
	0.86, 0.70-1.07	1.81, 1.40-2.34
	1.59, 1.23-2.04	

OR, odds ratio, adjusted for sex, age, smoking status, alcohol drinking, socioeconomic status, salt intake, vegetable and fruit intake
CI, confidence interval; NA, not available
* OR for ≤6 hours, 7 hours, and ≥9 hours (Reference category: 8 hours)
† OR for intermediate and high level (Reference category: low level)
